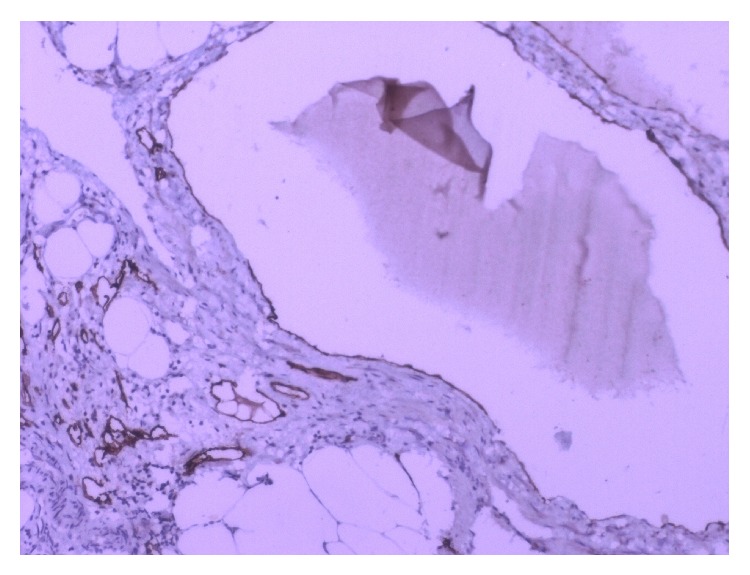# Retroperitoneal Lymphangioma in an Adult: A Case Report of a Rare Clinical Entity

**DOI:** 10.1155/2015/732531

**Published:** 2015-03-17

**Authors:** Mayank Tripathi, Sanjeev Parshad, Rajender Kumar Karwasra, Ashish Gupta, Saket Srivastva, Ankush Sarwal

**Affiliations:** ^1^Department of Surgical Oncology, Ward Nos. 6 and 8, Unit 1, Pt.B.D.Sharma, PGIMS, Rohtak, Haryana 124001, India; ^2^Department of Surgery, Pt.B.D.Sharma, PGIMS, Rohtak, Haryana 124001, India; ^3^Department of Surgery and Surgical Oncology, Pt.B.D.Sharma, PGIMS, Rohtak, Haryana 124001, India; ^4^Department of Urology, Pt.B.D.Sharma, PGIMS, Rohtak, Haryana 124001, India

## Abstract

*Background*. Retroperitoneal lymphangioma is a rare, benign mesodermal tumor arising from the retroperitoneal lymphatics which usually presents in infancy and it is worthy to report a case when it has presented in an adult. After a thorough literature search in English we concluded that less than 200 cases of adult retroperitoneal lymphangioma have been reported so far. Apart from being a rare entity it also presents as a diagnostic dilemma and final diagnosis is often made on surgical exploration. *Case Presentation*. We report a case of retroperitoneal lymphangioma in a 55-year-old male who presented with abdominal distension and dull aching abdominal pain. *Conclusion*. Retroperitoneal lymphangiomas are rare tumors of infancy but may also present in adults where they are a diagnostic challenge. Differentiating cystic lymphangiomas from other cystic growths by imaging studies alone are often inconclusive and surgery is frequently required for definitive diagnosis and to ameliorate the symptoms.

## 1. Introduction

Cystic lymphangioma is a rare benign, mesodermal tumor arising from lymphatic vessels. 75% of these lesions are present in the neck, 20% are in the axillary region, and only 5% are intraabdominal, where they have been reported in the mesentery, gastrointestinal tract, spleen, and liver, and very rarely in retroperitoneum. Retroperitoneal lymphangiomas account for less than 1% of all lymphangiomas [1, 2]. Retroperitoneal lymphangiomas are often asymptomatic and are usually detected intraoperatively or during imaging for some other conditions [3]. Less commonly when the cyst is large, patient may present with abdominal distension, pain, fatigue, and weight loss. A large cyst which undergoes torsion, hemorrhage, and rupture may present as acute abdomen. When presenting as a palpable abdominal mass they are easily confused with other cystic tumors including those arising from the liver, kidney, and pancreas. Imaging studies are often inconclusive in differentiating cystic lymphangiomas from other cystic lesions and surgery or diagnostic laparoscopy is most frequently required for definitive diagnosis and management [4]. Because of the rarity and the diagnostic dilemma that it poses, we are reporting a case of retroperitoneal cystic lymphangioma in a 55-year-old male.

## 2. Case Report

**Figure 1:**
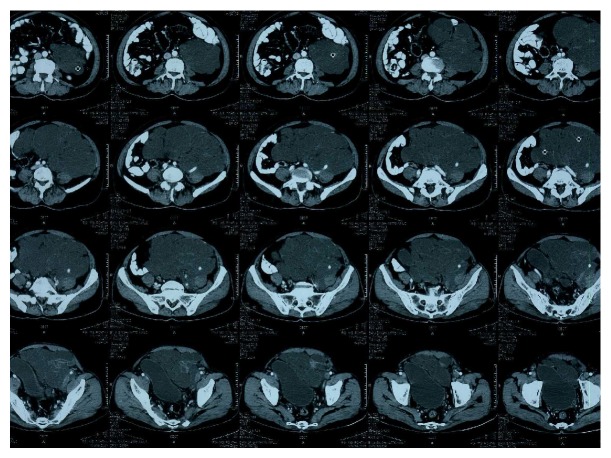


A 55-year-old man of Indian ethnicity, presented to the outpatient department of general surgery with lower abdominal distension for the past one and a half months and a continuous dull aching lower abdominal pain for past 15 days. Patient was a chronic smoker and gave no history of trauma, anorexia, weight loss, altered bowel habits, vomiting, fever, or any urinary symptoms such as hematuria. On examination, patient was moderately built, and abdominal fullness was apparent on inspection, more so in the left lower abdomen. A vague mass of approximate size of 20 × 15 cm was palpable, extending from left iliac and left hypogastrium to umbilical region, crossing the midline and reaching up to right midclavicular line at the level of iliac crest. The lump had a smooth surface, ill-defined margins and lower margin could not be reached on palpation. The lump was nontender with firm, cystic consistency, and restricted mobility in all the directions. There was no inguinal lymphadenopathy and digital rectal examination was unremarkable. Patient was further investigated for differential diagnoses of mesenteric cyst, pancreatic pseudocyst, duplication cyst, or cystic metastasis. Ultrasound abdomen showed a large cystic lesion with multiple septae, left to the urinary bladder in the pelvis. Liver, spleen, pancreas, and bilateral kidneys were within normal limits. A contrast enhanced computed tomography (CECT) scan of abdomen and pelvis showed a large 14.4 × 14.6 × 9.2 cm multilocular cystic mass with enhancing septations and lobulation, occupying the left side of abdominal cavity extending downwards into the pelvis and displacing the gut loops and the urinary bladder to the right side. Mass was encasing the left iliac artery and there were no enlarged lymph nodes in paraaortic and pelvic region. The attenuation coefficient of the mass was in the +10 to 15 HU range. There were no ascites and no evidence of small or large bowel obstruction (Figure 1).

A fine needle aspiration cytology of the mass showed RBCs, neutrophils, and lymphocytes in a proteinaceous background, entertaining the diagnosis of lymphangioma. A provisional diagnosis of retroperitoneal lymphangioma/mesenteric cyst was made and the patient underwent an exploratory laparotomy. Upon laparotomy, a large (15 × 15 cm), retroperitoneal multicystic mass was found, which was encasing the left common iliac bifurcation, left iliac vessels, left ureter, left gonadal vessels, and left vas deferens. The mass was pushing the urinary bladder towards right side. There was a plane of dissection among the vessels, ureter, urinary bladder, and the cyst, which was dissected free completely using sharp and blunt dissection. It was filled with approximately 1000 cc of thick, cloudy fluid. Frozen-section study of a portion of wall of the cyst was suggestive of lymphangioma. After removing the cyst completely, the retroperitoneum and the mesentery were inspected closely for any remnants. The bowel was inspected twice and was found to be healthy, viable, and without any evidence of involvement. The patient tolerated the procedure well and his postoperative course was uneventful.

Gross examination of the specimen, showed a mass of glistening white tissue and serial sectioning revealed multiple cysts filled with cloudy fluid.

**Figure 2:**
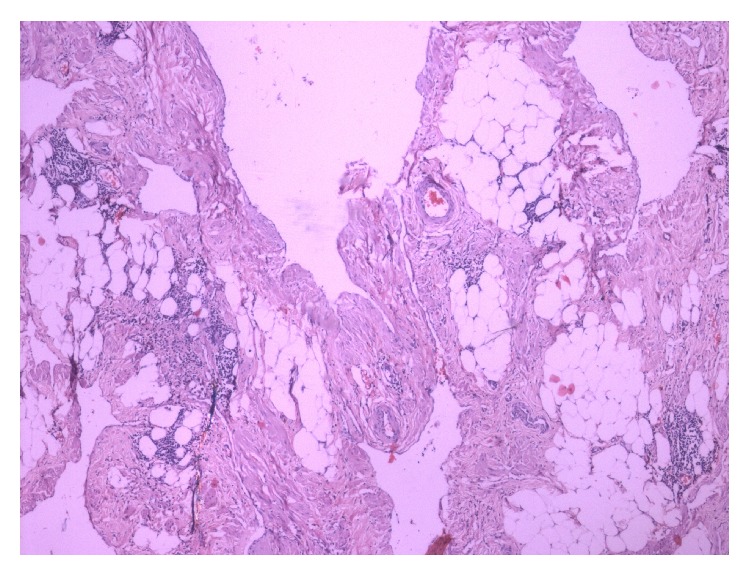


Histologically, the mass was composed of variable sized cystic spaces lined by flattened endothelial cells, which were positive for D2-40 immunostains, consistent with features of lymphatic vessels (Figures 2 and 3). The larger spaces had fascicles of smooth muscle, and nearly all of them were filled with pale pink proteinaceous material. Small lymphoid aggregates were present. The stroma showed acute on chronic inflammation, edema and fibrosis. Thus, the diagnosis of a retroperitoneal multilocular cystic lymphangioma was histologically confirmed. The patient has been on regular follow-up over the last one year and there has been no evidence of recurrence, clinically as well as on imaging.

## 3. Discussion

Our patient, a 55-year-old male, presented with a continuous dull aching pain and fixed lump in lower abdomen. A benign lesion was remotely a suspicion on clinical grounds because of his age and short duration of symptoms (2 months). Ultrasonography and CECT scan were suggestive of a multicystic lesion encasing iliac vessels and the possibilities of malignant mesothelial lesion, cyctic metastasis or a rare retroperitoneal lymphangioma were considered. FNAC did not reveal malignant cells and there was some amelioration of symptoms along with mild regression in size of lesion after the aspiration of about 100 mL of fluid. This regression in size was suggestive of a benign lesion. Surgical exploration revealed a multi-cystic lesion encasing iliac vessels, gonadal vessels, vas deferens and ureter. A clean plane of dissection could be demonstrated and all the structures were preserved while the whole lesion was excised in toto. Histopathology confirmed it to be lymphangioma. Diagnosis of lymphangioma of the retroperitoneum, in an adult, is a very rare clinical entity.

A lymphangioma is a benign proliferation of lymphatic tissue believed to originate from the early sequestration of lymphatic vessels that fail to establish connections with normal draining lymphatics at about 14–20 weeks of intrauterine life [5]. Lymphangiomas are therefore considered a congenital rather than an acquired tumor. After birth, they can become markedly dilated as a result of both the collection of fluid and the budding of preexisting spaces [6]. The other explanations for the origin of lymphangioma include obstruction of lymph channels secondary to fibrosis, inflammation, trauma, node degeneration; or failure of endothelial secretory function [7].

Lymphangiomas at retroperitoneal location are rare. Commonly accepted hypothesis regarding their origin is the development of abnormal connections between the iliac and retroperitoneal lymphatic sacs, and the venous system, leading to lymphatic fluid stasis in the sacs. In 1877, Wegner histologically divided lymphangiomas into three categories: (1) lymphangioma simplex (capillary lymphangioma) with small, thin-walled lymphatic channels and not commonly found intraabdominally; (2) cavernous lymphangioma with larger thin-walled channels, more common than lymphangioma simplex, but still rare intraabdominally, and may undergo malignant transformation; (3) cystic lymphangioma (always benign) composed of large cystic spaces lined with flat endothelium. Retroperitoneal lymphangiomas are usually of cavernous or cystic types, of which most reported cases have been of a cystic type, as was in our case [8].

A cystic tumor in the retroperitoneum creates a diagnostic confusion with varying differential diagnoses of cystic mesothelioma, teratoma, undifferentiated sarcomas like liposarcoma and leiomyosarcoma, metastatic lymphadenopathy, cystic metastases (especially from ovarian or gastric primaries), benign tumors such as lymphangioma, adenoma, and other tumors such as retroperitoneal hematoma, abscesses, duplication cysts, ovarian cysts and pancreatic pseudocysts [1].

Preoperative diagnosis of retroperitoneal lymphangioma is difficult. Ultrasound (US), contrast enhanced CT and MRI scans appear to be complementary to each other in the evaluation of cystic lymphangioma. US demonstrates the internal structure of lymphangiomas, particularly septations with clear fluid. CT may differentiate retroperitoneal and mesenteric lymphangiomas from adjacent bowel loops, and can also distinguish parapelvic renal lymphangiomatosis from hydronephrosis. In our case CT revealed a multi-loculated cystic mass with encasement of left iliac vessels. The ability of MRI scan to provide images in multiple planes without loss of resolution may demonstrate additional lesions and further delineate their boundaries [9]. Even with the availability of good imaging modalities, diagnosis of retroperitoneal lymphangioma is often made after laparotomy or laparoscopy and is confirmed by histopathological examination and immunohistochemistry [1].

Histological diagnosis of lymphangioma is based on well-established criteria [8]. These include a well-circumscribed, cystic lesion, with or without endothelial lining, a stroma composed of a meshwork of collagen and fibrous tissue, and a wall containing focal aggregates of lymphoid tissue. Histopathological examination of our case showed variable sized dilated cystic spaces lined by flattened endothelium, filled with pale pink proteinaceous material. Stroma showed acute and chronic inflammatory cells and evidence of fibrosis along with small lymphoid aggregates. Immunohistochemical markers used in the diagnosis of lymphangioma are lymphatic vessel endothelial receptor-1, vascular endothelial growth factor-3, monoclonal antibody D2-40 and prox-1. In our case the endothelial lining of the cyst showed positive reaction for D2-40 antibody and CD31.

Surgical excision in totality is the treatment of choice because of its potential to grow and invade surrounding organs [10]. Complete surgical excision is often difficult to achieve because of local invasiveness which may lead to encasement of structures like major vessels and ureter. Incomplete excision often leads to recurrence and redo surgery is quite challenging [11]. In our case, even though preoperative imaging was suggestive of encasement of left iliac vessels, on surgical exploration these could be dissected free from the lymphangioma and a complete surgical excision was achieved. Retroperitoneal dissemination during surgery is very rare but potentially fatal [12]. Hauser et al. suggested that isolation and ligation of the cystic lymphangioma's peduncle as well as ligation of lymph channels can prevent recurrences and chylascos [13]. Although marsupialization, aspiration, drainage, and irradiation of the lymphangioma have been described but they give a poor result and are not recommended [1]. Treatment by argon beam ablation and sclerotherapy has also been reported in a patient with a life-threatening total abdominal lymphangiomatosis [14].

At one year follow-up our patient was completely free of any disease on clinical and radiological examination.

## 4. Conclusion

Retroperitoneal lymhangioma is a rare clinical entity in adults. Differentiating cystic lymphangiomas from other cystic growths by imaging studies alone is often inconclusive and surgery is most frequently required for definitive diagnosis and to ameliorate the symptoms. It is possible to completely excise the lesion even if it is seen to encase major vessels as a clean plane of dissection usually exists.

## Figures and Tables

**Figure 3:**